# Rasch analysis of the General Self-Efficacy Scale in a sample of persons with morbid obesity

**DOI:** 10.1186/1477-7525-11-202

**Published:** 2013-11-25

**Authors:** Tore Bonsaksen, Anders Kottorp, Caryl Gay, May Solveig Fagermoen, Anners Lerdal

**Affiliations:** 1 Department of Occupational Therapy, Prosthetics and Orthotics, Faculty of Health Sciences, Oslo and Akershus University College of Applied Sciences, Oslo, Norway; 2 Department of Neurobiology, Care Sciences and Society, Karolinska Institutet, Stockholm, Sweden; 3 Lovisenberg Diakonale Hospital, Oslo, Norway; 4 Lovisenberg Diakonale University College, Oslo, Norway; 5 Department of Nursing Science, Institute of Health and Society, Faculty of Medicine, University of Oslo, Oslo, Norway

**Keywords:** General self-efficacy scale, Morbid obesity, Rasch analysis, Psychometrics, Patient education

## Abstract

**Background:**

Self-efficacy is needed for effectuating lifestyle changes, and it is therefore an important target related to health. The purpose of this study was to evaluate the psychometric properties of the General Self-Efficacy Scale (GSE) using Rasch analysis in a sample of adults with morbid obesity.

**Methods:**

A convenience sample of adults with morbid obesity was recruited from patient education courses. A total of 141 participants completed the GSE and a demographic questionnaire at the beginning of the course. The statistical approach included analysis of rating scale function, item fit to the Rasch partial credit model, unidimensionality, aspects of person-response validity, person-separation reliability, and differential item function. A version omitting items with poor fit to the Rasch model was also evaluated.

**Results:**

The rating scale did not advance monotonically for item #2 in the original 10-item version, and the first three GSE items did not demonstrate acceptable goodness-of-fit to the Rasch model. In a 7-item version omitting these three items, the rating scale functioned well for all items, and all items demonstrated good fit to the Rasch model. Both the 10-item and 7-item versions of the GSE partially met the criteria for unidimensionality. Neither version met the criterion for person response validity, although the results were slightly better for the 7-item than for the 10-item version. Both versions of the GSE demonstrated the ability to separate the respondents into three distinct levels of general self-efficacy. Several items had differential item function in relation to age, education or work status, but there were fewer in the 7-item version.

**Conclusions:**

For adults with morbid obesity, a 7-item version of the GSE seems to have better psychometric properties than the original 10-item version.

## Background

Obesity has become a major public health problem, and the proportion of the population with morbid obesity (body mass index > 40) is increasing [1]. Morbid obesity is a risk factor for chronic somatic illnesses such as diabetes, hypertension, heart disease, stroke, and cancer [2-4]. In addition, obesity is related to lower psychological well-being and quality of life [5,6]. Lifestyle factors are considered important contributors to the condition. Most directly, they concern the pattern of nutrition and physical activity in people’s lives. Indirectly, they also include a wide range of activities, such as seeing a friend for social support instead of over-eating as a means of reducing emotional stress [7].

Because of the health risks associated with obesity, it is important to support persons with obesity in making lifestyle changes. In order to achieve a more health-promoting lifestyle, a person needs to believe that he or she can perform the behaviors that lead to better health. Such beliefs are referred to as self-efficacy and concern a person’s beliefs about how capable he or she is in performing the behaviors needed to bring about the desired outcome. Those who believe that they can achieve what they set forth to do tend to stick to their plan, invest adequate energy and effort in their actions, and do not easily give up when experiencing setbacks [8]. Furthermore, the importance of self-efficacy is not limited to behavior change – empirical studies have found that self-efficacy also predicts distal outcomes like health and quality of life in illness groups as diverse as chronic obstructive pulmonary disease [9], arthritis [10], and heart disease [11].

Given the many-faceted context of activities, relationships, and cultural influences with which persons with morbid obesity have to cope, assessing their self-efficacy *only* for specific activities like exercise and dieting appears to be a limited approach. Assessing the person’s self-efficacy for coping with challenging activities and situations in general may be equally important, and it has been proposed that this generalized sense of competence can predict a complex set of health perceptions and -behaviors [12]. For persons with morbid obesity, health perceptions may concern the perceived ability to exert control over one’s own health situation, and not consider oneself a victim of illness. Discrete health behaviors may include dieting and physical activity, but also seeking advice and support from others when needed. In support of this reasoning, one study of adolescent girls at risk for excessive weight gain found that higher general self-efficacy was associated with fewer episodes of uncontrolled eating and with lower total intake at the meal [13]. Moreover, a weight loss intervention study with overweight and obese adults showed weight loss in the participants after treatment that was sustained at six months follow-up, with increasing levels of general self-efficacy during the same period [14]. These findings not only highlight the relevance of general self-efficacy for persons with morbid obesity, but also point to the importance of having valid measures for assessing general self-efficacy and evaluating interventions for this population.

The *General Self-Efficacy Scale* (GSE) [15] has become a widely used instrument for measuring general self-efficacy. The GSE assesses “a broad and stable sense of personal competence to deal effectively with a variety of stressful situations” [12; p.3]. It consists of 10 items that are rated on a scale from 1 (“not at all true”) to 4 (“exactly true”). The ten items of the GSE are listed in Table 1.

**Table 1:** Items of the general self-efficacy scale

**Item #**	**Item description**
1	I can always manage to solve difficult problems if I try hard enough
2	If someone opposes me, I can find the means and ways to get what I want
3	It is easy for me to stick to my aims and accomplish my goals
4	I am confident that I could deal efficiently with unexpected events
5	Thanks to my resourcefulness, I know how to handle unforeseen situations
6	I can solve most problems if I invest the necessary effort
7	I can remain calm when facing difficulties because I can rely on my coping abilities
8	When I am confronted with a problem, I can usually find several solutions
9	If I am in trouble, I can usually think of a solution
10	I can usually handle whatever comes my way

*Note.* All items have the following response format: 1 = not at all true, 2 = hardly true, 3 = moderately true, 4 = exactly true [15].

The GSE sum score is calculated by summing the item scores, and ranges between 10 (lowest GSE) and 40 (highest GSE). The scale has been used in research with college students [16-18] and population cohorts [19], as well as with clinical populations, including persons with breast cancer [20], renal disease [21], morbid obesity [22,23] or chronic obstructive pulmonary disease [22]. High correlations between GSE and a range of social-cognitive variables, including behavior-specific self-efficacy, indicate theoretical accuracy of the general self-efficacy concept [16,24]. Factor analysis of the GSE has consistently produced a one factor solution; that is, only one underlying dimension has been found [25]. In Norway, Leganger and colleagues [26] found GSE item-total correlations ranging between 0.25 and 0.63, with factor loadings ranging between 0.32 and 0.74 and internal consistency (Cronbach’s α) = 0.82. Recent research has also used modified versions of the GSE in research with pulmonary rehabilitation patients [27] and patients at risk for heart failure [28]. In the former case, five items specifically related to the management of chronic obstructive pulmonary disease were added to the GSE. In the latter case, four items (items 1, 6, 8, and 9) were removed from the GSE in order to test the psychometric properties of a shorter version of the scale. These later efforts highlight the need to examine the psychometric properties of the GSE in light of the specific populations with which it is used. Most of the above-cited studies, however, have examined the properties of the GSE by means of classical test theory (CTT) approaches. These approaches assume interval data and that all items in a scale are equally difficult. In addition, CCT does not allow for a separation of the evaluated persons and items – these are both interpreted in the context of the other. On the other hand, modern psychometric approaches, like Item Response Theory (IRT), estimate each item’s difficulty as well as each person’s ability on the same metric, allowing for meaningful comparisons of the two. Moreover, they examine each item’s relationship to the measured theoretical construct [17].

The Rasch model, which is one specific application of IRT, has been used for decades in order to analyze ordinal data in order to provide linear measures, by the use of logarithmic transformation procedures [29]. A Rasch-based analytical approach generates reliability and validity estimates of both persons and items that are independent of the sample distribution. These estimates can be used for in-depth monitoring of test functioning. For example, GSE items and persons demonstrating poor fit to the Rasch model have unexpected response patterns given the item’s estimated difficulty and the respondent’s estimated level of general self-efficacy. This information can be useful in identifying items that do not contribute to a valid measure of the underlying trait, as well as potential response biases related to respondent characteristics, such as age or gender.

The literature review performed for this study identified two recent studies using an item response theory approach to assess the psychometric properties of the GSE [28,30]. However, only the study by Peter and colleagues [30] used the Rasch analysis method. In their study, the GSE was used with a sample of persons with spinal cord injury (*N* = 102), and they concluded that the data fitted a unidimensional construct [30]. Each item fit the unidimensional Rasch model, and the items functioned in a similar way across age, gender, education levels, and functional limitations, with no evidence of significant differential item functioning. Person reliability was high, and the GSE was also able to separate participants into five distinct levels of self-efficacy. In conclusion, the GSE was found to function as a psychometrically sound measure of self-efficacy for persons with spinal cord injury, but with a ceiling effect – generally, self-efficacy levels among the participants were higher than reflected by the GSE items. To date, it appears that no studies available have used a Rasch analysis approach to examine the psychometric properties of the GSE in persons with morbid obesity.

### Purpose

A one-year prospective longitudinal study was designed to explore changes in health-related quality of life in persons with morbid obesity and persons with chronic obstructive pulmonary disease, and also to test 12 instruments with regard to their ability to detect change over time [23]. The purpose of this article is to report on the psychometric properties of the GSE in a sample of persons with morbid obesity.

### Aims and research questions

The aim of this study was to examine and evaluate evidence of validity of the GSE when used with persons with obesity. The research questions for this study were:

1. What is the structure of the GSE response scale, and more specifically, do the response categories [1 = not at all true; 2 = hardly true; 3 = moderately true; 4 = exactly true] logically reflect less/more self-efficacy in persons with morbid obesity?

2. Do the items in the GSE support a unidimensional underlying construct; that is: (a) Do the GSE items’ response patterns across the participants demonstrate acceptable goodness-of-fit to the Rasch model (i.e., do the items exhibit expected response patterns given each participant’s estimated level of general self-efficacy)? (b) Is the majority of the variance explained by a single underlying construct?

3. Do the participants’ response patterns on the GSE items demonstrate acceptable goodness-of-fit to the Rasch model (i.e., do the participants exhibit expected response patterns given each item’s estimated level of difficulty)?

4. Does the GSE separate the sample into a sufficient number of distinct levels of general self-efficacy?

5. Are item difficulty calibrations stable in relation to age, sex, work status, education level, and relationship status (differential item functioning, DIF)?

## Methods

### Sample

Participants were recruited in 2009 from 10 patient education courses at three different sites in Oslo and the surrounding areas in south-eastern Norway. Patients were referred to the course by their physician, as the course is a mandatory requirement for persons with morbid obesity who want to be considered for bariatric surgery. The inclusion of each participant required the person to have a body mass index of 40 kg/m^2^ or greater [31]. All 185 participants attending the courses were given verbal and written information about the study and invited to participate in the study on the first day of the course. Those who gave their written consent completed the study questionnaire in a secluded room on-site and returned it in a sealed envelope. The project representative collected the envelopes.

### Instruments

Participants completed the Norwegian version of the GSE [32] (described above) and a demographic questionnaire, which collected data about the participant’s age (years), sex, marital status (married/cohabitant *versus* not married/not cohabitant), and employment status. Participants’ formal level of education was dichotomized as 12 years (secondary) education or less *versus* more than 12 years (university/college) education.

### Statistical analysis

A two-faceted (item and person) Rasch partial credit model was applied to the GSE data. The Rasch model takes each item scored and adjusts the final person measure based on relative differences in item difficulty. Rasch models are also suitable for handling data where item scores may be missing. Although only a small number of item scores were missing across the 141 participants, i.e., 11 item scores out of 1410 (0.8%), we did not have to exclude any participant or item scores due to missing data [33-35].

The WINSTEPS analysis software program, version 3.69.1.16 [36] was used to perform all Rasch analyses. The Rasch analysis converts the raw item scores into equal-interval measures using a logarithmic transformation of the odds probabilities of responses. The converted values are then used to examine whether the items from the scale measure a single unidimensional construct, a psychometric property viewed as crucial in both classical and modern measurement statistics [33,37]. The logarithmic transformation simultaneously results in an estimation of each person’s level of general self-efficacy as well as the difficulty of each item (i.e. targeting lower to higher levels of self-efficacy) along a calibrated continuum. A Rasch partial credit model was applied to the GSE in this sample, as the generic scale used in GSE may not function in a similar manner across all items.

First, we evaluated the functioning of the GSE rating scale, according to the following criteria: a) the category measures on each item should advance monotonically. In order to judge category measures, the average measure was used as an indicator, and b) a criterion less than 2.0 was expected in outfit mean square *(MnSq)* values for each item response category calibration [38]. If the response categories do not advance monotonically, collapsing response categories is suggested to minimize this problem [38].

After the analysis of the rating scale, we proceeded with the analysis by evaluating (1) the fit of the GSE items, (2) the unidimensionality of the GSE, (3) person-response validity, (4) the ability of the GSE to separate people into distinct levels of general self-efficacy (sensitivity of the GSE scale), and (5) the stability of item hierarchy across key demographic variables, by assessing uniform differential item functioning (DIF).

Evidence of *internal-scale validity* (1) and *person-response validity* (3) were investigated using goodness-of-fit statistics using the WINSTEPS program to generate mean square *(MnSq)* residuals and standardized *z-*values for both items and persons. These values indicate the degree of match between actual responses on the GSE and expected responses from the Rasch model. The goodness-of-fit statistics were evaluated using *infit* statistics. *Infit statistics* are information-weighted fit statistics that give relatively more weight to the performances of persons who are well targeted to the item difficulty calibrations. As *infit* statistics are more informative when exploring the fit of items to the Rasch model and person response validity [39,40], we chose *infit* statistics to evaluate goodness-of-fit across items and across persons in this study.

The *MnSq* fit statistic has an expected value of 1.0 and is preferable for item goodness-of-fit with polytomous data (as in the GSE) as it is less sensitive to sample size compared to *z*[41]. We chose to use a sample-size adjusted criterion for *item goodness-of-fit* which accepted infit *MnSq* values between 0.7 and 1.3 logits [41].

The criterion for evaluating *person goodness-of-fit* was to accept infit *MnSq* values ≤ 1.4 logit and/or an associated *z* value < 2 [42,43]. It is generally accepted that 5% of the sample, by chance, may not demonstrate acceptable goodness-of-fit without a serious threat to person-response validity [42,43], and thus we chose this proportion as a guideline for our analysis of person goodness-of-fit in the GSE. We also monitored the targeting of the GSE in relation to the current sample by evaluating how many participants generated a higher or lower measure than the highest and lowest item threshold (i.e., where the probability is 50/50 of giving each of two scores). The proportion of this sample beyond the thresholds indicates the proportion of participants for whom the GSE version is not adequately targeted or sensitive (ceiling and floor effects).

To evaluate unidimensionality of the GSE, and thus minimize the risk of additional explanatory factors in the measures generated, a *principal component analysis* (PCA) of residuals was performed [44]. Two criteria were used: a) at least 50% of the total variance should be explained by the first latent variable/dimension, and b) any additional factor should explain < 5% of the remaining variance of after removal of the first latent variable/dimension [45,46]. We also evaluated the item residual correlations in order to monitor local independence, with a criterion set than not more than 5% of the item residual correlations should exceed 0.30 [47].

To further determine whether the GSE scale could distinguish people with different levels of general self-efficacy, *person-separation reliability* was assessed. For clinical purposes, we chose a criterion that the scale should be able to distinguish at least three groups (indicating high, medium, and low levels of general self-efficacy), which requires a *person separation index* of at least 2.0 [48]. For the purpose of comparison to more traditional reliability estimates, the Rasch-equivalent Cronbach’s alpha statistic was also assessed.

We finally performed a number of uniform DIF analyses to evaluate the stability of the GSE item calibrations across key demographic variables (age, gender, work, education, and relationship status). These variables were selected based on their potential to influence GSE. The magnitude of DIF was evaluated using the Mantel-Haenszel statistic for polytomous scales using log-odds estimators [49,50] in the WINSTEPS program [51]. Although a Bonferroni correction yielding a 1% alpha is commonly used [51], we also report results with *p* < 0.05 to more conservatively evaluate the likelihood of item bias. We expected that not more than 5% of the potential item DIF iterations should demonstrate significant DIF.

Initially, an analysis of all ten GSE items was performed. If an item did not demonstrate acceptable goodness-of-fit to the model according to the set criteria, one item at a time was removed and psychometric properties were re-analyzed with the remaining items. This procedure was repeated until all items demonstrated acceptable goodness-of-fit. After each item removal, unidimensionality, person response validity, and reliability of the GSE measures were re-evaluated as described above.

SPSS for Windows Version 19.0 software was used to analyze demographic data. Descriptive statistics were used to summarize the sample characteristics, and independent sample *t*-tests were used to compare demographic groups.

### Ethics

The Norwegian Research Ethics Committee and the Ombudsman of Oslo University Hospital approved of the study (REK S-08662c 2008/17575). Informed written consent was obtained from all participants. The study is registered in Clinical Trials: NCT01336725.

## Results

### Sample characteristics

Of the 185 individuals invited to participate in the study, 142 (76.8%) consented. One participant who did not complete any of the 10 GSE items was excluded. The socio-demographic characteristics of the 141 participants included in the analysis are presented in Table 2. The male participants were older than the female participants (45.5, SD 9.2 *versus* 41.1, SD 10.7, *t*[139] = 2.28, *p* = .024), but no other sex differences were found. Participants and non-participants did not significantly differ with respect to age or sex (data not shown).

**Table 2:** Demographic characteristics of the sample and GSE scores (N = 141)

	**Demographic characteristic**	**GSE scores mean ( *SD *)**
Full sample, range 12 – 39		26.5 (6.3)
Age (years), range 20 – 60	42.4 (10.4)	
< 40 years	*n* = 57 (40%)	26.7 (6.5)
≥ 40 years	*n* = 84 (60%)	26.4 (6.3)
Sex		
Male	*n* = 41 (29%)	27.0 (6.1)
Female	*n* = 100 (71%)	26.3 (6.5)
Relationship status (*n* = 140)		
Not in paired relationship	*n* = 48 (34%)	26.3 (6.1)
In paired relationship	*n* = 92 (66%)	26.6 (6.5)
Education level		
≤ 12 years	*n* = 95 (67%)	26.2 (6.5)
> 12 years	*n* = 46 (33%)	27.2 (6.0)
Employment status (*n* = 140)		
Not working	*n* = 63 (45%)	26.9 (6.1)
Working	*n* = 77 (55%)	26.1 (6.7)

*Note.* All *p*-values > .05.

### Rating scale functioning

In the first step, the functioning of the rating scale was examined, and some items were found to have fewer than ten observations in the extreme response categories (1 and 4). Items #1, #6, #8 and #9 had less than ten responses in category 1, while item #3 had less than ten responses in category 4. Since the category measures advanced monotonically in each of these items, we did not consider the limited number of responses in these categories to be a significant threat to the validity of the GSE. However, for item #2 the category measures on the GSE rating scale did not advance monotonically. On this item, response categories 1 (not at all true) and 2 (hardly true) were reversed, and thus, categories 1 and 2 were collapsed for item #2 in all subsequent validity analyses.

### The fit of the items to the Rasch model

In the second step, analysis of the content validity of the ten GSE items revealed that item #2 did not demonstrate acceptable goodness-of-fit to the Rasch model, meaning that the participants’ scores on this particular item were inconsistent with their overall response patterns. The analysis therefore continued by removing that item and repeating the analysis on the remaining nine items in GSE. Subsequent iterations also removed items #3 and #1 (see Table 3), and the seven remaining items all demonstrated acceptable goodness-of-fit. The items, measures, and item fit statistics of the resulting GSE-7 are shown in Table 4.

**Table 3:** Overview of the analytic process using a Rasch model approach

**Step**	**Psychometric property**	**Statistical approach and criteria**	**Results original 10-item GSE**	**Results reduced 7-item GSE (omits items with poor fit)**^**a**^
1	**Rating scale functioning:** Does the rating scale function consistently across items? (substantive validity)	• Average measures for each step category and threshold on each item should advance monotonically	• Rating scale met criteria for all items but item 2. Scale steps 1 and 2 reversed. Recoded into 1(2)34scale	• Rating scale met criteria
		• *z*-values < 2.0 in outfit mean square *(MnSq)* values for step category calibrations^b^		
2	**Internal scale validity:** How well do the actual item responses match the expected responses from the Rasch model? (content validity)	Item goodness-of-fit statistics • *MnSq* values < 1.3^c^	• 3 items failed to meet criterion^d^: • Item 2: *MnSq*=1.64 (1) • Item 3: *MnSq*=1.39 (2) • Item 1: *MnSq*=1.38 (3)	• All items met criterion
3	**Internal scale validity:** Is the scale unidimensional (i.e., does it measure a single construct)? (structural validity)	Principal component analysis • ≥ 50% of total variance explained by first component (general self-efficacy)^e^ • Any additional component explains < 5% (or eigenvalue<2.0) of the remaining variance after removing first component^e^ No more than 5% (or 1 out of 20) of the residual correlations >.30	• First component explained 61.3% of total variance • Second component • explained 6.9% of total variance, but eigenvalue <2.0 (1.8) • One out of 45 (2.2%) residual correlations >.30 (#3 - #8: *r* = -.31)	• First component explained 64.5% of total variance • Second component explained 8.9% of total variance, but eigenvalue <2.0 (1.7) • One out of 21 (4.8%) residual correlations >.30 (#4 - #9: *r* = -.33)
4	**Person-response validity:** How well do the individual responses match expected responses from the Rasch model? (substantive validity)	Person goodness-of-fit statistics • *Infit MnSq* values < 1.5 and *z-*value ≤ 2.0^f^ • ≤ 5% of sample fails to demonstrate acceptable goodness-of-fit values^f^	• 13/14 respondents (9.2/9.9% of sample) failed to demonstrate acceptable goodness-of-fit values	• 8/9 respondents ( 5.7/6.4% of sample) failed to demonstrate acceptable goodness-of-fit values
		• *Infit MnSq* values < 1.5 and *z-*value ≤ 2.0^f^		
		• ≤ 5% of sample fails to demonstrate acceptable goodness-of-fit values^f^		
5	**Person-separation reliability:** Can the scale distinguish ≥3 distinct groups of self-efficacy in the sample tested? (reliability)	Person-separation index • ≥ 2.0^g^	• 2.75	• 2.67
6	**Internal consistency:** Are item responses consistent with each other? (reliability)	Cronbach’s alpha coefficient • > 0.8^g^	• 0.93	• 0.93

*Note.* After initial evaluation of the original 10-item GSE, a stepwise process was used whereby items failing to meet criteria were removed one at a time, and only those meeting criteria in earlier steps advanced to subsequent steps. If more than one item failed to meet a criterion, the item with the worst fit was removed and the step was repeated with the remaining items. The last column includes ^a^ 7-item version omitting misfitting items 1, 2, and 3.

^b^[38].

^c^[52].

^d^Items are listed in the order of removal and the *MnSq* values shown reflect the iteration prior to the item’s removal.

^e^[47].

^f^[53].

^g^[48].

**Table 4:** Items, measures, and item statistics of the 7-item version of the General Self-Efficacy Scale

**Item #**	**Item description**	**Item measure (logits)**	**Item fit statistics**
7	I can remain calm when facing difficulties because I can rely on my coping abilities	60.65	1.00
5	Thanks to my resourcefulness, I know how to handle unforeseen situations	55.99	0.89
4	I am confident that I could deal efficiently with unexpected events	54.47	0.86
10	I can usually handle whatever comes my way	53.17	1.17
8	When I am confronted with a problem, I can usually find several solutions	49.44	1.00
9	If I am in trouble, I can usually think of a solution	40.55	0.86
6	I can solve most problems if I invest the necessary effort	35.72	1.14

*Note.* Lower measures = higher scores; higher measures = lower scores. Items are in decreasing level of difficulty.

### Unidimensionality

In the third step, we examined unidimensionality of the scale to determine whether it measures a single underlying construct. The PCA for the different GSE versions is presented in Table 3. The Rasch model explained between 60.4% and 64.3% of the variance across the different iterations, with the highest explained variance in the 7-item version. These proportions met the criterion for the first dimension, but the secondary dimension explained between 7.1% and 9.0% of the total variance, which were slightly higher than the expected 5%. When evaluating the item residuals of the GSE 7 item version, only one out of 21 correlations (4.7%) was above 0.3, further supporting local independence of the items and unidimensionality. The evidence of unidimensionality was therefore mixed in each of the GSE versions; however, the 7-item version demonstrated the highest degree of explained variance and local independence, and it was the only version in which all items demonstrated acceptable goodness-of-fit.

### Person-response validity

The fourth step of the analysis examined person-response validity. Of the 141 GSE surveys, 13 (9.2%) failed to demonstrate acceptable goodness-of-fit to the Rasch model in the 10-item version, indicating that the response patterns of these persons were unlikely given their underlying level of general self-efficacy. This proportion was reduced slightly as the misfitting items were removed, with the 7-item GSE having 8 respondents (5.7%) failing to demonstrate acceptable goodness-of-fit. There were no systematic demographic differences between the respondents with and without misfit. Thus, we concluded that the GSE demonstrated a somewhat higher level of misfit among participants than expected, although the 7-item version was slightly better than the 10-item version.

**Figure 1:**
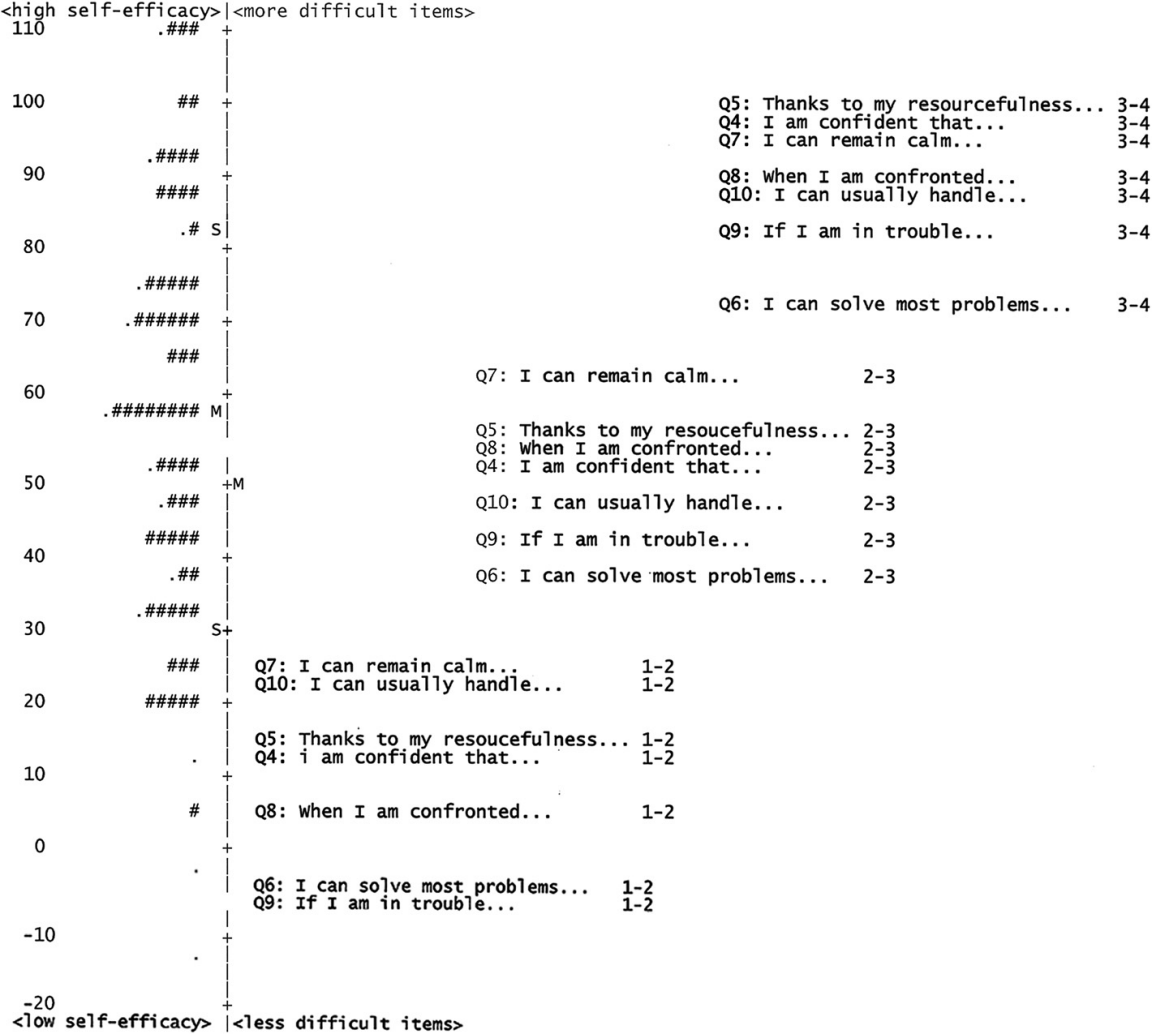
**Person-Item map for the 7-item version of the GSE.** Legend: A person-item map of the GSE-7 items in a sample of people with morbid obesity (n = 141). Each item threshold is presented in the table, where there is a 50/50 chance of giving any of the stated scores. The mean (M) and standard deviation (SD) of the sample is presented, in relation to the item mean (set at 50 by default). Each "#" represents two persons and each "." represents one person.

To monitor the targeting of the GSE in relation to the current sample, the number of participants with maximum and minimum scores across the different GSE-item solutions was evaluated, as shown in Table 2. None of the participants had a maximum score on the 10-item version, but 5 (3.5%) respondents had a maximum score on the 7-item version. None of the participants obtained a minimum score on either version of the scale. When specifically evaluating how many of the participants scored higher or lower than the item thresholds, eight participants (5.7%) were outside the maximum range of the GSE 7-item version (seven above and one below the maximum range). The distribution of the sample in relation to the item thresholds are presented in Figure 1. There was also a difference between the sample mean (*M* = 58.6, 95% confidence interval [CI] = 54.0-63.2) and item mean (*M* = 50.0, 95% CI = 43.4-56.6), indicating that overall the sample had higher self-efficacy than the GSE item target.

### Person separation reliability and internal consistency

The fifth step of the analysis examined the GSE in terms of its ability to separate persons into groups based on different levels of general self-efficacy. The person separation index was 2.75 for the original 10-item version of the GSE and 2.67 for the 7-item version, indicating that both versions can detect three statistically distinct groups of participants within the sample. The Rasch-equivalent Cronbach’s alpha coefficient for all of the GSE versions was 0.93. The distributions of persons and GSE items (and each item threshold per response category) for the 7-item version are presented in Figure 1.

### Differential item functioning (DIF)

Table 5 shows how the GSE rating scales – both the original version and the 7-item version – functioned across age, sex, work status, education level, and relationship status. The GSE items in both 10- and 7-item versions functioned similarly in relation to participant sex and relationship status. However, in the 10-item version, item #1 functioned differently by age group, and items #2 and #4 functioned differently by education level. Item #7 functioned differently in relation to work status in both the 10-item and 7-item versions. In the 7-item version, item #8 also demonstrated DIF in relation to education level. When the significance level was adjusted for the number of comparisons (*p* < 0.01) none of the DIFs were significant in the 10-item version, and only one (item #7) had significant DIF in the 7-item version.

**Table 5:** Differential item functioning in the original and the reduced GSE versions

**Differential item functioning**	**Results**	**Results**
**(DIF):** Are item difficulty calibrations stable in relation to the following demographic variables? (generalizability validity)	Original 10-item GSE	Reduced 7-item GSE (omits items with poor fit)^a^
Age	Item 1: easier to agree with for people < 40 (p = .045)	• No DIF
Gender	• No DIF	• No DIF
Work	• Item 7: easier to agree for workers (p = .012)	• Item 7: easier to agree for workers (p = .003)
Education	• Item 2: easier to agree for persons with higher education (p = .045)	• Item 8: easier to agree for higher education (p = .046)
	• Item 4: easier to agree for persons with lower education (p = .024)	
Relationship	• No DIF	• No DIF

*Note*. One item with DIF out of 20 can be expected to occur by chance and is deemed acceptable. Thus, the criterion for differential item function was a Mantel-Haenszel statistic [49] with *p* < 0.01 after Bonferroni correction [51]. Using an uncorrected *p*-value of < 0.05 is not common, but minimizes the risk of underestimating item bias.

^a^Items #1, 2, and 3 removed.

## Discussion

This study was the first to examine the psychometric properties of the *General Self-Efficacy Scale* in a sample of persons with morbid obesity using a Rasch analysis approach. The original 10-item version explained 61.3% variance by the first latent dimension (general self-efficacy), but three items demonstrated poor fit to the Rasch model and were deleted. The resulting 7-item GSE functioned reasonably well in this sample, and a higher variance proportion (64.5%) was explained by the first dimension in this version. However, the variance explained by the second dimension exceeded the criterion of 5% for both versions of the scale, suggesting the possibility of a minor second dimension. There were also limitations related to person response validity, as both versions exceeded the criterion of <5% of persons demonstrating unacceptable fit to the Rasch model. Both versions were sufficiently sensitive to be able to distinguish three distinct groups of participants. The good separation ability also enhances the measure’s sensitivity for detecting change.

Items #1 and #2 both demonstrated misfit to the Rasch model. This may indicate a general misfit of these items across sample populations, and not a particular misfit among persons with morbid obesity, given that a study on spinal cord injury patients found similar problems with these items [30]. Item misfit essentially indicates that the respondents rated this item inconsistently in relation to their overall response pattern. For item #1, the misfit may be explained by different interpretations of the item. A person emphasizing the latter part of the item (“…if I try hard enough”) would perhaps be able to give a high rating to this item, even if he or she had low scores on the other items. Conversely, a person with high ratings on the other items may still have given item #1 a low rating if he or she emphasized the first part of the item (“I can always manage to solve difficult problems…”).

For item #2, the misfit may be explained by this item being the only one to include an interpersonal aspect. For participants with otherwise equal levels of general self-efficacy, some felt a strong sense of competence in getting what they want in spite of others opposing them, whereas others felt they had less such competence. Previous research has shown that persons who are obese experience prejudice and discrimination [54]; thus, some of the participants may have had difficult experiences with others opposing them, whereas others have not. Given the similarities between our results and those of Peter and colleagues [30], an alternative explanation is that item #2 does not function well as part of the GSE scale in general, and is not specifically problematic among persons with morbid obesity.

With respect to item #3, it may be that the sample characteristics played a significant part in determining some participants’ response. In the context of a diagnostically targeted patient education course, some may have rated item #3 with their health condition specifically in mind. That is, their interpretation of the phrase “it is easy for me to stick to my aims and accomplish my goals” may have been related to their goal of losing weight and living healthier in terms of what they eat and how physically active they are in their daily life. On the other hand, others may have more general aims and goals in mind when responding to this item. This discrepancy may have led to item #3 misfitting with the GSE scale.

It could be argued that it is not a good idea to remove an item which seems particularly relevant for the obesity population. In particular, item #3 may represent a core struggle for persons who are obese. From a clinical point of view, it has been emphasized that persons with morbid obesity may be quite knowledgeable about what they should do and not do in order to improve their lifestyle – what is often lacking is the ability to persist in doing what is needed to achieve a healthier lifestyle [7,22]. However, if this item has the potential of being interpreted very differently between respondents, it may not fit well with the *general* self-efficacy construct being assessed. So the item may still generate clinically important information regarding the person with morbid obesity, but will not fit the measurement scale. Persistence in sticking to aims and accomplishing goals in the specific context of obesity management should preferably be addressed in specific measures of self-efficacy for targeted behaviors and activities that are particularly relevant for persons with morbid obesity.

Removing the three misfitting items will make the sum score of the remaining items difficult to compare to previous research results. It is also noteworthy that the previously constructed short version of the GSE yielded a different solution, including items 2, 3, 4, 5, 7, and 10 in the resulting scale [28]. If future studies with participants with morbid obesity use the GSE-7, as suggested by this study, the scores can be adjusted to correct for the reduced number of items, but comparisons to 10-item scores, and in particular to shorter scales composed of different items, should be made with caution.

The study also showed lower GSE levels in this sample of participants with morbid obesity than what has been previously shown with large normal population samples [25]. It makes sense that having a chronic health problem, like morbid obesity, can be associated with lower self-efficacy. A health problem may in and of itself diminish the person’s view of him- or herself as someone who can deal with important challenges in life, and especially so when the health problem is viewed as closely related to the person’s own lifestyle, as is the case with morbid obesity. Failing to follow what the person knows to be an effective course of action, like adhering to a diet or increasing physical activity, may detract from the person’s self-efficacy.

On the other hand, age may be related to self-efficacy. In a large Norwegian study, general self-efficacy in a sample of 18-year old adolescents (Mean GSE = 24.3) was substantially lower than in a sample of adult smokers who were about 40 years of age (Mean GSE = 29.6) [26]. The difference was discussed in light of Bandura’s social cognitive theory [55], suggesting that adolescence is a period of transition requiring the mastery of new skills as the person gradually progresses into adult age. Comparing our sample to the two subsamples in Leganger’s study [26], our sample had a mean age similar to that of the subsample of adult smokers, but had GSE levels similar to those of the subsample of adolescents. Attending a patient education course indicates making an effort to change lifestyle, and as such, it may also indicate an uneasy period of transition. Thus, the lower levels of GSE in our sample may be partly explained by their unstable situation and the stress they may experience during the process of change. This comparison may also speak to a larger impact of stress in determining the level of self-efficacy, as compared to the impact of age.

The targeting between the GSE items and the participants in this study indicated that the GSE items target lower levels of self-efficacy than generally possessed by the sample, as evidenced by the higher person means compared to item means. On the other hand, the 95% CI of the means are overlapping, and only 5.7% of the sample scored outside the range of the GSE 7-item version, and the majority of those scored higher. Given these findings, we suggest that the GSE targeting is acceptable for the sample tested. It may also be more important to target those with lower levels of self-efficacy than to differentiate between those with higher levels. From a clinical point of view, it is those with lower levels of self-efficacy that may need additional support and intervention.

### Study strengths and limitations

The main strength of this study is the use of a modern test theory approach – Rasch analysis – to investigate the psychometric properties of the GSE, an instrument widely used in research related to health and quality of life. In addition, the study was based on a sample of persons with morbid obesity with a high participation rate and relatively little missing data, thereby minimizing the likelihood of bias. Furthermore, evaluating the psychometric properties of both the original GSE and a version omitting misfitting items allowed for the direct comparison of the two versions.

A limitation of this analysis is that it is based on a fairly small sample, and this needs to be considered in the interpretation of the results. Even though the overall sample size in this study can generate relatively precise item calibrations [56], the DIF findings are more speculative as the sizes of the subgroups are smaller than optimal. The fact that we were able to distinguish three levels of general self-efficacy also suggests that relatively robust parameters were obtained. All participants were recruited from patient education courses, and thus, this sample may differ from the broader population of persons with morbid obesity. Attending such a course may indicate that the participants were highly motivated to improve their health condition. Furthermore, the overall patterns of general self-efficacy in this sample may differ from those of persons who are not attending such courses. This ambiguity concerning the participants’ motivation for making changes and the possibility of different patterns of general self-efficacy in this group may limit the generalizability of these results.

## Conclusion and directions for future research

The original 10-item GSE functioned as a unidimensional measure, but three items showed poor fit to the applied Rasch model. After removal of these items (#1, #2, and #3), the resulting 7-item version showed better psychometric properties than the original. However, in cases where it is relevant to examine self-efficacy beliefs item by item, it may be appropriate to use all 10 items, despite the fact that three items showed poor fit to the Rasch model in this study. Given that few research studies have used a Rasch analysis approach to examining the GSE, conducting Rasch analytic studies of the scale with various groups of people appears to be one line of future psychometric research on the GSE. Eventually, such efforts may lead to modifications of the scale for the specific groups it is used with.

## Competing interests

The authors declare that they have no competing interests.

## Authors’ contributions

TB drafted the manuscript. AK performed the statistical analysis and contributed to the drafting of the manuscript. CG contributed to the drafting of the manuscript. MSF designed the study, collected the data, and contributed to the drafting of the manuscript. AL contributed to the design of the study and contributed to the drafting of the manuscript. All authors read and approved the final manuscript.
